# Implementation and Initial Analysis of a Laboratory-Based Weekly Biosurveillance System, Provence-Alpes-Côte d’Azur, France

**DOI:** 10.3201/eid2304.161399

**Published:** 2017-04

**Authors:** Michael Huart, Gabriel Bedubourg, Cédric Abat, Philippe Colson, Jean Marc Rolain, Hervé Chaudet, Pierre Edouard Fournier, Didier Raoult, Xavier Deparis

**Affiliations:** Centre d’Epidémiologie et de Santé Publique des Armées, Marseille, France (M. Huart, G. Bedubourg, X. Deparis);; Unité de Recherche sur les Maladies Infectieuses et Tropicales Emergentes, Aix-Marseille Université, Marseille (M. Huart, C. Abat, P. Colson, J.M. Rolain, H. Chaudet, P.E. Fournier, D. Raoult);; Fondation Institut Hospitalo-Universitaire Méditerranée Infection–Assistance Publique-Hôpitaux de Marseille, Marseille (M. Huart, C. Abat, P. Colson, J.M. Rolain, P.E. Fournier, D. Raoult);; Sciences Economiques et Sociales de la Santé et Traitement de l’Information Médicale, Aix Marseille Université, Marseille (M. Huart, C. Abat, P. Colson, J.M. Rolain, P.E. Fournier, D. Raoult)

**Keywords:** infectious diseases, microbiology laboratories, epidemiologic surveillance system, epidemiology, biosurveillance, Provence-Alpes-Côte d’Azur, PACA, PACASurvE, France, bacteria

## Abstract

We describe the implementation of an automated infectious disease surveillance system that uses data collected from 210 microbiologic laboratories throughout the Provence-Alpes-Côte d’Azur region in France. Each week, these facilities report bacterial species that have been isolated from patients in their area. An alarm is triggered whenever the case count for a bacterial species infection exceeds 2 SDs of the historical mean for that species at the participating laboratory. At its inception in July 2013, the system monitored 611 bacterial species. During July 1, 2013–March 20, 2016, weekly analyses of incoming surveillance data generated 34 alarms signaling possible infectious disease outbreaks; after investigation, 14 (41%) of these alarms resulted in health alerts declared by the regional health authority. We are currently improving the system by developing an Internet-based surveillance platform and extending our surveillance to include more laboratories in the region.

During the second half of the 20th century, infectious diseases were considered a public health concern belonging to the past (*1*). However, despite some decrease in epidemiologic importance (*2*), infectious diseases remain a major cause of illness and lead to >25% of annual deaths (*3*–*5*). To ensure the timely detection of infectious diseases, health authorities have proposed the implementation of health surveillance systems. Historically, surveillance started with the use of mortality and morbidity data for public health purposes, which was first proposed by John Graunt in 1657 (*6*). The concept of surveillance has evolved over the centuries, and surveillance is now conducted mainly through the monitoring of symptoms and syndromes. During the 20th century, an expansion of the surveillance concept occurred with the emergence of numerous surveillance systems (*4*,*7*). Epidemiologic surveillance came to be known for 3 basic characteristics: systematic collection of data, consolidation and analysis of the collected data, and dissemination of information through narrative epidemiologic reports (*3*). Since 2001, because of the threat of bioterrorist attacks and the emergence and reemergence of infectious diseases, such as the recent Ebola outbreak in West Africa, interest in the methods for detection of infectious diseases has increased (*4*,*8*).

In the Assistance Publique-Hôpitaux de Marseille (AP-HM) public hospital network in Marseille, France, weekly automated epidemiologic surveillance systems have been implemented since 2002 (*9*,*10*). The objectives of these systems are to analyze clinical data produced by the microbiologic laboratories of 4 public hospitals in Marseille. The first program implemented, the Epidemiologic Surveillance and Alert Based on Microbiological Data, has monitored more than 293 infectious disease–related items on a weekly basis since November 2002 (*9*), including 38 clinical samples, 86 pathogens, 79 diagnosis tests, and 39 antimicrobial-resistance patterns. After the introduction of this system, several other systems based on a previously described historical database (*11*) were set up, such as the Bacterial Real-Time Laboratory-Based Surveillance System (BALYSES) and the Marseille Antibiotic Resistance Surveillance System (MARSS) (*10*). The latter 2 systems have routinely operated in the AP-HM network since 2013. During May 21, 2013–June 4, 2014, BALYSES detected 21 alarms (triggered when the number of cases of an infectious disease exceeds the statistic threshold), and MARSS detected 31. For BALYSES, 5 alarms either were escalated into alerts after further investigation or led to official reports to the Regional Health Agency (Agence Régionale de Santé [ARS]) of PACA, and for MARSS, 16 alarms led to official reports (*10*).

In July 2013, we aimed to expand the epidemiologic surveillance implemented in the AP-HM network to the entire PACA region by developing a new specific surveillance tool. This tool was developed in collaboration with the Armed Forces Center for Epidemiology and Public Health in France (CESPA). Until that time, no laboratory network in France had been implemented to monitor so many infectious disease–related events (*12*). Several other epidemiologic surveillance networks of microbiologic laboratories exist worldwide, such as the system set up by C. Paddy Farrington et al. in England and Wales (*13*) or the “Vigie” network in Belgium (*14*). 

The PACA region is a population hub with many airports and ports with large flows of migrants and travelers. These population flows could bring infectious disease into the PACA region. Therefore, implementation of such a biosurveillance system based on previously unused data from microbiologic laboratories in the PACA region was expected to improve infectious diseases surveillance. Here we describe the procedure for implementing this biosurveillance system in and the initial results obtained from July 2013 through the end of March 2016.

## Implementing a Biosurveillance System

### Study Setting

**Figure 1:**
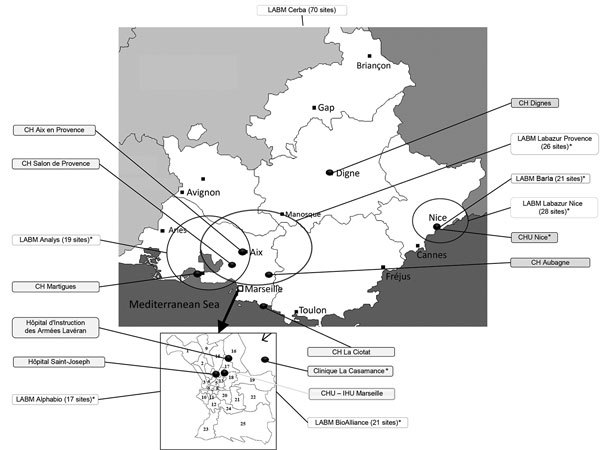
Laboratories participating in the Provence Alpes Côte d’Azur Surveillance Epidemiologic System, France, July 1, 2013–March 20, 2016. Black dots indicate participating laboratories; black boxes indicate public laboratories; text labels indicate private laboratories and areas of activity. Asterisks (*) denote laboratories using matrix-assisted laser desorption/ionization time-of-flight mass spectrometry for identification of species; all other laboratories shown use biochemical bacterial identification. CH, Centre Hospitalier (Central Hospital); CHU, Centre Hospitalier Universitaire (Central University Hospital); IHU, Institut Hospitalo-Universitaire (Hospital–University Institute); LABM, Laboratoire de Biologie Médicale (Medical Laboratory).

The PACA region is located in southeastern France and is the third most populated region in the country, with ≈4.9 million inhabitants (≈7.5% of the total French population) (*15*). Several major cities are located in the region, such as Marseille, Toulon, and Nice, with 850,636, 163,974, and 343,064 inhabitants, respectively, in 2014 (*15*). The PACA region, which borders on Italy, is an important hub, with large population flows from North Africa across the Mediterranean Sea. In 2015, the PACA region had ≈611 private or public microbiologic laboratories according to ARS (ARS, unpub. data). Some of these laboratories have formed groups or networks, which can consist of up to 70 laboratories (Table 1). The geographic coverage of the laboratories included in our biosurveillance network is homogeneous over the region (Figure 1).

**Table 1:** Selected characteristics of facilities participating in the Provence Alpes Côte d’Azur Surveillance Epidemiologic System, France, July 1, 2013–March 20, 2016*

Facility	Status	Geographic area	No. laboratories, N = 210	Wks since launch	Continuous or discontinuous	Start date of surveillance
LABM Labazur Provence	Private	Bouches-du-Rhône, Var, Vaucluse	26	142	Continuous	2013 Jul 1
LABM Alphabio	Private	Marseille	17	142	Continuous	2013 Jul 1
Clinique La Casamance	Public	Marseille	1	139	Continuous	2013 Jul 22
LABM Analys	Private	Boûches-du-Rhône	19	127	Discontinuous	2013 Oct 14
CH Aix-en-Provence	Public	Aix-en-Provence	1	122	Discontinuous	2013 Nov 18
CHU Nice	Public	Nice	1	118	Continuous	2013 Dec 16
CH Martigues	Public	Martigues	1	114	Continuous	2014 Jan 13
CH Salon-de-Provence	Public	Salon-de-Provence	1	109	Discontinuous	2014 Feb 14
Hôpital Inter-Armées, Laveran	Public	Marseille	1	100	Discontinuous	2014 Apr 21
LABM Cerba	Private	PACA	70	63	Continuous	2015 Jan 5
Hôpital Saint-Joseph	Public	Marseille	1	59	Discontinuous	2015 Feb 3
LABM BioAlliance	Private	Marseille	21	42	Continuous	2015 Jun 1
LABM Labazur Nice	Private	Alpes Maritimes	28	23	Continuous	2015 Oct 12
CH Dignes	Public	Dignes	1	11	Discontinuous	2016 Jan 1
LABM Barla	Private	Nice	21	7	Continuous	2016 Feb 3

*CH, Centre Hospitalier (Central Hospital); CHU, Centre Hospitalier Universitaire (Central University Hospital); LABM, Laboratoire de Biologie Médicale (Medical Laboratory); PACA, Provence Alpes Côte d’Azur.

### Biosurveillance System

We created a biosurveillance system, the PACA Surveillance Epidemiologic System (PACASurvE), capable of collecting, standardizing, and computing the laboratory results produced by public (i.e., hospital-affiliated) and private-sector microbiology laboratories located in the PACA region every week. The system’s objectives are to provide early detection capability and an initial description of possible infectious disease threats (*10*,*16*); accordingly, the system is designed to issue alarms if an outbreak is detected or if a single case of a rare but severe infectious disease or an unknown infectious agent is discovered.

PACASurvE is Internet-based and uses Excel software (Microsoft, Redmond, WA, USA) for data collection and management and R version 3.0.1 software (*17*) for analysis. The system was implemented and has been routinely used since July 2013. Public hospital and private sector laboratories of the PACA region were invited to participate in the surveillance network. Fifteen institutions were first selected among the leading laboratories in terms of volume of activity to rapidly achieve a better geographic representation of PACA (Figure 1). Their participation was based on several criteria proposed in the literature and relevant to the implementation of our network, including those described by Walckiers et al. (*14*): participation of laboratories on an unpaid and voluntary basis, participation of microbiologic laboratories, anonymity of data, and a standard, predefined frequency for data collection (*14*).

After identifying the participating institutions, the second task was to define which events should be monitored and their respective definitions, which were transmitted to all laboratories. Data collected included information on bacterial identification and virologic, bacteriologic, mycologic, and parasitologic laboratory results. Currently, PACASurvE is particularly geared toward the monitoring of bacterial species.

We defined a case as illness in a patient from whom >1 bacterial species was isolated and confirmed. Two different bacterial species isolated from samples collected from the same patient resulted in 2 reported cases. The samples without bacterial identification were considered to be negative. Laboratories were free to use the microbiologic methods of their choice to identify bacterial species, including PCR, matrix-assisted laser desorption/ionization time-of-flight mass spectrometry, or conventional phenotypic methods (e.g., Gram coloration and API galleries). 

**Figure 2:**
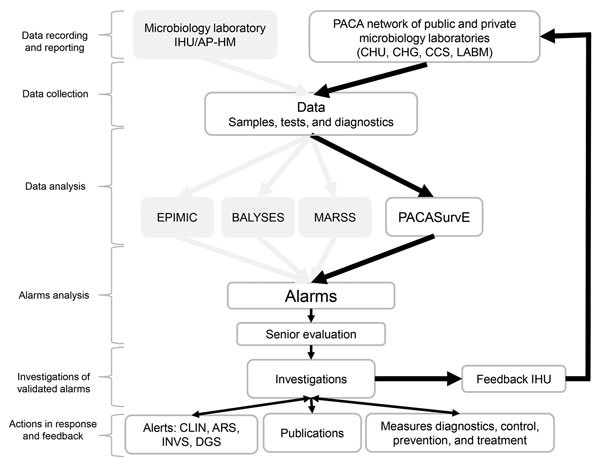
Flow diagram of all epidemiologic surveillance systems implemented by the Institut Hospitalo-Universitaire Méditérannée Infection, Assistance Publique-Hôpitaux de Marseille, France. ARS, Agence Régionale de Santé (Regional Health Agency); BALYSES, Bacterial Real-Time Laboratory-Based Surveillance System; CDS, Centre de Santé (Health Center); CHG, Centre Hospitalier Général (General Hospital Center); CHU, Centre Hospitalier Universitaire (Central University Hospital); CLIN, Comité de Lutte contre les Infections Nosocomiales (Committee for the Fight Against Nosocomial Infections); DGS, Direction Générale de la Santé (Directorate General for Health); EPIMIC, Epidemiologic Surveillance and Alert Based on Microbiological Data; IHU/AP-HM, Institut Hospitalo-Universitaire/Assistance Publique-Hôpitaux de Marseille; INVS, Institut Nationale de Veille Sanitaire (National Institute for Public Health Surveillance); LABM, Laboratoire de Biologie Médicale (Medical Laboratory); MARSS, Marseille Antibiotic Resistance Surveillance System; PACASurvE, Provence Alpes Côte d’Azur Surveillance Epidemiologic System. Diagram is based on the workflow described by Abat et Al. 2013 (*10*).

A weekly coordination meeting, which included members from AP-HM and CESPA, was defined to optimize data collection and organize analyses and feedback. Currently, PACASurvE is included in a comprehensive biosurveillance system at the AP-HM network with the other epidemiologic surveillance systems previously described (Figure 2).

### Data Flow, Analyses, and Feedback

All the steps of data flow, analyses, and feedback were mapped (Figure 2). Every week, biologists at participating institutions sent a report of new cases, in the form of anonymized data contained in Excel spreadsheets or encrypted PDFs, to the system coordinator.

The first step before analysis was validating and standardizing the reported data, which were checked, cleaned, and deduplicated. The search for duplicates was performed weekly according to the unique patient identifier and the isolated microorganism, and data were then automatically compared with a thesaurus of all identified bacterial species (*10*). All analysis steps (e.g., deduplication, merging of data from different laboratories, statistical analysis, and visualization tools production) were performed automatically by using a specific algorithm written in a Visual Basic (Microsoft) script for Excel.

To detect outbreaks as early as possible, an alarm was triggered when the weekly count of cases for a bacterial species was higher than 2 SDs of the mean of historical data since the beginning of surveillance for each laboratory (*10*). After 6 months, once the collection procedures were stabilized, the C1-mild epidemics detection method used by the Early Aberration Reporting System (*18*,*19*) was performed. This method enables the detection of outbreaks on a dataset with limited historical data (>7 weeks). Both of these statistical methods operate in parallel at the Institut Hospitalo-Universitaire Méditérannée Infection and CESPA.

A statistical alarm is triggered if the observed value is significantly different from the expected value (*16*). After checking biologic criteria, alarms were assessed as confirmed or unconfirmed by senior biologists during the weekly AP-HM epidemiologic surveillance meeting. Alarms that were escalated into an alert (after further investigations that included diagnosis confirmation and descriptive analysis of cases in terms of time, place, and population) led to further epidemiologic investigation, which then had to be declared to ARS if a real outbreak was confirmed. Specific countermeasures also had to be implemented, such as patient isolation, implementation of specific care protocols, or a large scale information campaign (Figure 2). For feedback, a weekly epidemiologic report was addressed to all participating laboratories, AP-HM department officials, CESPA, ARS, and the Interregional Epidemiology Unit (otherwise known as CIRE).

## Results

### Scalability of the System

In July 2013, when PACASurvE started, 3 main structures that collected data from 44 laboratories sent their anonymized data to the network coordinator every Monday. In March 2016, a total of 15 participating institutions were included in the biosurveillance system (Table 1). Several participated irregularly; 2 (Centre Hospitalier Dignes and Centre Hospitalier Aix en Provence) transmitted a common declaration file. Currently, PACASurvE includes 8 public and 7 private sector participating institutions (Figure 1), representing a total of 210 laboratories (34.4% of all laboratories in the PACA region).

### Description of Collected Cases

An average of 14,000 cases (positive and negative) were reported every week. Since the beginning of the biosurveillance system, 217,621 bacterial infections have been reported by participating structures (i.e., ≈1,532 confirmed cases per week). These identifications resulted from the analysis of an estimated 315,000 urine samples, 140,000 blood cultures, 6,700 respiratory specimens, 32,000 stool samples, 4,400 cerebrospinal fluid samples, and 176,000 serologic examinations.

### Thesaurus

Data were compared automatically to a thesaurus that included 611 bacterial species at the time of the system’s inception in 2013 (*10*). Currently, the number of bacterial species is 673.

### Top 10 Identified Bacteria in PACASurvE and Biodiversity

We ranked the overall top 10 bacterial species isolated since the beginning of the biosurveillance system and the top 10 per laboratory. The 10 most frequently reported bacterial species in PACASurvE represented 181,241 identifications (83.1% of total cases) (Technical Appendix). *Escherichia coli* infections were the most frequently reported cases in all the laboratories. For others species, diversity increased when frequency decreased (Technical Appendix).

### Bacterial Species Specifically Isolated by PACASurvE

PACASurvE has also made it possible to identify bacterial species that were unknown in the initial thesaurus of AP-HM (*10*). A total of 12 bacterial species have been isolated and added to this thesaurus: *Citrobacter werkmanii, Kluyvera cryocrescens, Lactobacillus brevis, Streptococcus pluranimalium, Paenibacillus peoriae, Rhodotorula minuta, Cronobacter malonaticus, Paenibacillus durus, Rhodotorula mucilaginosa, Rhizobium radiobacter, Buttiauxella agrestis,* and *Plesiomonas shigelloides*.

### Alarms and Alerts

The biosurveillance system issued alarms every week after analysis. These alarms were triggered by an increasing number of reported cases for some bacterial species. The system has issued 5,915 alarms since July 2013, averaging 2,160 alarms per year and 41 alarms per week. Since July 2013, after analysis at the weekly coordination meetings, 34 alarms required further investigations after validation by a senior epidemiologist or a biologist, and 14 (41%) of those were escalated to an alert. We ranked the 10 bacterial species that have triggered the largest number of alarms since the beginning of the surveillance network (Table 2).

**Table 2:** Ten bacterial species with the most alarms triggered by the Provence Alpes Côte d’Azur Surveillance Epidemiologic System, France, July 1, 2013–March 20, 2016*

Bacterial species	No. alarms total	Average weekly no. alarms
*Pseudomonas putida*	87	0.6
*Stenotrophomonas maltophilia*	82	0.6
*Neisseria gonorrhoeae*	78	0.6
*Hafnia alvei*	74	0.5
*Enterobacter aerogenes*	72	0.5
*Staphylococcus capitis*	72	0.5
*Staphylococcus lugdunensis*	70	0.5
*Streptococcus constellatus*	68	0.5
*Staphylococcus haemolyticus*	66	0.5
*Haemophilus parainfluenzae*	65	0.5

*Total no. alarms for the entire system during this period was 5,915 (averaging 42 alarms weekly).

Fourteen notifications have been reported to ARS as outbreaks, which were caused by the following bacterial species: *Enterococcus faecalis*, *Clostridium difficile* (serotype O27 and non-O27), *Escherichia coli, Acinetobacter radioresistens, Serratia marcescens, Enterobacter aerogenes, Proteus penneri, Streptococcus pyogenes,* and *Streptococcus aerogenes.* All these epidemiologic events were identified by PACASurvE and confirmed after further detection by other surveillance systems. Other alarms were declared for laboratories or hospitals involved in investigations. These investigations did not necessarily lead to an alert but more often to an internal investigation.

Alarms regarding an *E. faecalis* outbreak were issued by PACASurvE in March 2015 (*20*), largely because of an increase in the number of declared cases of *E. faecalis* isolated in urine samples in Marseille and surroundings areas. That outbreak was reported to ARS, and investigations are still ongoing to find out if a single or multiple clones were responsible.

Alarms regarding *C. difficile* were issued in the hospital monitoring system BALYSES and among private-sector laboratories in the PACASurvE system. These alarms led to a further investigation into *C. difficile*–related illness and death in patients. We found the presence of a hypervirulent strain (O27) (*21*). However, this strain does not account for all the deaths attributable to *C. difficile* (*22*) because other strains of this bacterial species exist. Therefore, an alert was issued to ARS, which led to specific countermeasures (e.g., systematic screening, isolation of patients, transport to the infectious diseases unit at Hôpital Nord in Marseille, and establishment of a specific treatment protocol with early fecal transplant) (*23*).

### Feedback and Network Management

The feedback bulletin was set up to keep all participants in the surveillance network informed. It consisted of a presentation with 2–3 slides per participating laboratory, with a summary of their declared activity during the previous week. It was accompanied by an email newsletter with information on the main alarms that led to further investigations and interpretation by a college of experts. The weekly epidemiologic bulletin was also available on the website of the Institut Hospitalo-Universitaire Méditérannée Infection (http://www.mediterranee-infection.com/article.php?larub=23&titre=surveillance-epidemiologique).

## Discussion

Since July 2013, we have been operating a biosurveillance system based on a network of clinical microbiology laboratories in the PACA region to monitor infectious diseases, especially those attributable to bacterial species.

To date, to our knowledge, the PACASurvE network is unique in France. It is a collaborative system (one relying on the participation of private and hospital laboratories) which is different from BALYSES (a hospital system) (*10*). Similar biosurveillance networks were developed in Belgium (*14*,*24*), the United Kingdom (*13*), and the United States, where the Laboratory Response Network was implemented by the Centers for Disease Control and Prevention in 1999 (*25*). In Belgium, a sentinel network using microbiology laboratory data was created for the weekly monitoring of selected pathogens (*24*,*26*). In contrast, PACASurvE focuses on the monitoring of 673 bacterial species.

In the United States, the Laboratory Response Network was implemented to build a network of laboratories that can respond to biologic and chemical emergencies. Our surveillance system only focused on biologic threats and was intended for the early detection of infectious disease outbreaks on a weekly basis.

After initial difficulties in enrolling laboratories, the biosurveillance system now functions regularly. The system has generated 14 alarms that have been investigated and reported to ARS. These alarms made it possible to detect actual outbreaks and helped to develop effective countermeasures, as in the case of the *C. difficile* (*23*) and *E. faecalis* (*20*) outbreaks.

This system has several strengths and some limitations. The first and main strength of the system is that it can easily be replicated, thanks to its low implementation cost and its use of Excel software. The use of this software allowed the system to be set up rapidly, and any necessary modifications can be made easily, compared with the software used by other surveillance systems, such as Real Time Outbreak and Disease Surveillance (*27*). This simplicity could allow it to be implemented in developing countries.

Continuous improvement also is a major strength of PACASurvE. The number of laboratories increases regularly, which improves the representativeness of the PACA region. Improving geographic representativeness is important for the purposes of extrapolating our results or extending the system to other regions. Currently, the coverage of the system includes 214 laboratories in the PACA region, representing 81% of major urban areas of the PACA region (e.g., Marseille, Nice, and Toulon). Only 1 department of the region, the Hautes Alpes, is not properly covered.

The third strength is the diversity of bacterial species identified and transmitted by the network of laboratories in the PACA region. The number of samples tested and their diversity are greater in the PACASurvE than in other surveillance systems currently active in France (*10*). This difference could be explained by the higher number of participating laboratories, which increases continually. This high diversity underlines the relevance of a private-sector laboratory surveillance system operating in parallel with a hospital epidemiologic surveillance system. Currently, PACASurvE monitors only bacterial species, but it would be interesting to extend this surveillance to other subjects, such as antibiograms or viruses.

Our biosurveillance system has some limitations. The first relates to the statistical analyses used at the beginning for the detection of abnormal events. The use of a threshold of 2 SDs higher than the historical weekly mean is not necessarily appropriate, although it is easy to set up rapidly and enabled detection of an abnormal event. At the beginning of the surveillance system data collection, in the absence of strong historical data, this basic algorithm seemed to be the most appropriate and easy to use given the circumstances. After studying the methods used to address seasonal variations and sporadic emergence of rare bacterial species as described by Enki et al. (*13*) in 2013, Farrington et al. (*28*) in 1996, Buckeridge et al. (*29*) in 2004, and Frickers (*30*) in 2008, we decided to implement another method and this introduced the C1-mild epidemics detection method (*18*) with R software into the surveillance package. This method is now used routinely in CESPA.

The second limitation concerns the laboratories’ willingness to participate, which could lead to problems in reporting. For example, we have to encourage laboratories to report their data automatically by using the Internet platform.

After only 2 years in operation, the results achieved by our network are already promising. The economic cost of this system will be calculated, being a major criteria for the first planned evaluation of the system. In the future, we will improve the completeness of transmitted data and will try to extend our network to other regions in France. This type of regional biosurveillance network could be linked to data from existing networks implemented by the National Institute for Public Health Surveillance (Santé Publique France) to enable comprehensive surveillance of all French territory. An equivalent of the Epidemiologic Surveillance and Alert Based on Microbiological Data system has been set up in Senegal with the participation of several health centers. In conclusion, the recent development of a surveillance network based on data from microbiologic laboratories in the PACA region has demonstrated its value for early identification of regional epidemics.

Technical Appendix Summary of the 10 bacterial species most frequently isolated per participating institution since the beginning of their participation in the Provence Alpes Côte d’Azur Surveillance Epidemiologic System, France, July 1, 2013–March 20, 2016.
